# Damaged mitochondria coincide with presynaptic vesicle loss and abnormalities in alzheimer’s disease brain

**DOI:** 10.1186/s40478-023-01552-7

**Published:** 2023-03-31

**Authors:** Wenzhang Wang, Fanpeng Zhao, Yubing Lu, Sandra L. Siedlak, Hisashi Fujioka, Hao Feng, George Perry, Xiongwei Zhu

**Affiliations:** 1 Department of Pathology, Case Western Reserve University, 2103 Cornell Road, Cleveland, OH 44106 USA; 2 Cryo-EM Core Facility, Case Western Reserve University, Cleveland, OH USA; 3 Department of Population and Quantitative Health Sciences, Case Western Reserve University, Cleveland, OH USA; 4 Department of Neuroscience, Developmental and Regenerative Biology, University of Texas, San Antonio, TX USA

**Keywords:** Alzheimer disease, Mitochondria, Synapse, Dense core vesicle, Dendritic spine, Synaptic vesicles

## Abstract

Loss of synapses is the most robust pathological correlate of Alzheimer’s disease (AD)-associated cognitive deficits, although the underlying mechanism remains incompletely understood. Synaptic terminals have abundant mitochondria which play an indispensable role in synaptic function through ATP provision and calcium buffering. Mitochondrial dysfunction is an early and prominent feature in AD which could contribute to synaptic deficits. Here, using electron microscopy, we examined synapses with a focus on mitochondrial deficits in presynaptic axonal terminals and dendritic spines in cortical biopsy samples from clinically diagnosed AD and age-matched non-AD control patients. Synaptic vesicle density within the presynaptic axon terminals was significantly decreased in AD cases which appeared largely due to significantly decreased reserve pool, but there were significantly more presynaptic axons containing enlarged synaptic vesicles or dense core vesicles in AD. Importantly, there was reduced number of mitochondria along with significantly increased damaged mitochondria in the presynapse of AD which correlated with changes in SV density. Mitochondria in the post-synaptic dendritic spines were also enlarged and damaged in the AD biopsy samples. This study provided evidence of presynaptic vesicle loss as synaptic deficits in AD and suggested that mitochondrial dysfunction in both pre- and post-synaptic compartments contribute to synaptic deficits in AD.

**Supplementary Information:**

The online version contains supplementary material available at 10.1186/s40478-023-01552-7.

## Introduction

Alzheimer’s disease (AD) is characterized by progressive cognitive decline and affects 5.8 million people over the age of 65 in the United States alone [1]. This neurodegenerative condition can also affect younger individuals, referred to as early onset AD, where the clinical symptoms appear before the age of 65 [1]. Neuropathological diagnosis includes examination of the brain tissue after autopsy looking for the presence of extracellular amyloid plaques as well as neuron loss and neurodegenerative lesions including neurofibrillary tangles and granulovacuolar degeneration [1]. Unfortunately, there is no cure for this neurodegenerative condition and the only therapeutics available have limited effect on slowing the disease progression [1]. Thus, understanding the earliest events in the disease as well as developing novel targets for future therapeutics is of vital importance.

Synaptic function underlies cognition and synaptic abnormalities and dysfunction during Alzheimer’s disease have long been documented in the literature [13, 17]. Using confocal imaging, transmission electron microscopy and 3-dimension (3D) electron microscopy (EM), studies of human brain, including cortical, transentorhinal cortex, hippocampus and cerebellar regions, found there is a loss of synapses in AD [4, 17, 19, 21, 60, 74]. Biochemical studies confirmed that there is a striking loss of presynaptic vesicle proteins (synaptophysin and Rab3A, as examples) in hippocampal and cortical regions of AD brain [16, 55, 65, 71, 72]. Furthermore, synaptic loss can be detected in patients with mild cognitive impairment (MCI), a prodromal stage of AD, suggesting that it occurs early during the course of AD [42]. In fact, among all the early changes, synaptic loss is the most robust correlate of AD-associated cognitive deficits [12, 19, 74], leading to the notion that synaptic dysfunction plays a critical role in the pathogenesis of AD [13, 62]. However, mechanisms underlying synaptic loss and dysfunction remains elusive.

Synapses are the highest energy-consuming sites in the brain dependent on ATP mainly supplied by mitochondria to maintain ionic gradients and neurotransmission events [40]. Acutely or chronically inhibiting the respiratory chain causes a drop in mitochondrially-derived ATP which impairs synaptic transmission and/or synaptic growth/morphology [41, 47]. Mitochondria also provide calcium buffering capacity [34] critical for synaptic function such as synaptic vesicle (SV) recycling [66]. Therefore, it is imperative for mitochondria to travel from the soma to the extremities at the end of axons and dendrites to meet synaptic energetic and calcium buffering needs locally [40]. In fact, synaptic terminals have abundant mitochondria and these synaptic mitochondria differ in size, trafficking mechanisms, proteomic profiling, functional efficiency and lifespan from non-synaptic mitochondria [9, 25]. Electron microscopy studies revealed that synaptic terminals with the highest energy demands contained the greatest volume of mitochondria [33]. The paucity of mitochondria leads to the loss of dendritic spines and synapses [39, 76], suggesting their indispensable role at these sites.

Mitochondrial dysfunction is an early and prominent feature of AD [69, 78]. Severe mitochondrial abnormalities including changes in morphology, mitochondrial swelling, and loss of internal cristae structure, are found in pyramidal neurons in the brain of AD patients [29, 80]. Axonal defects that consist of swellings due to abnormal accumulations of motor proteins, organelles, and vesicles are found in the brain of AD patients [68] which likely deplete mitochondria from axons or more remote sites of neuronal processes in AD brain, suggesting that mitochondrial dysfunction could underlie synaptic abnormalities in AD. It was demonstrated that amyloid-β (Aβ) significantly interfered with mitochondrial dynamics and distribution and caused changes in both presynapse and spines in cell models of AD [22, 56, 77, 79–81]. Moreover, synaptic mitochondria showed greater alterations and Aβ accumulation [22] and there was selective regional loss of cortical synapses lacking presynaptic mitochondria in AD mouse models [63]. While regional loss of mitochondria in presynaptic terminals has also been reported in cortical regions of AD autopsy brain samples [49], the use of autopsied brain samples limited detailed analysis on synaptic and mitochondrial changes and their relationship. Using a library of EM images of a series of cortical brain biopsy samples from AD and non-AD patients, we sought to examine mitochondrial deficits in presynaptic axonal terminals and dendritic spines and correlate these deficits with synaptic defects to better understand the contribution of mitochondrial deficits to synaptic abnormalities in the pathogenesis of AD.

## Methods

### Tissue imaging

Archived samples of plastic-embedded brain biopsy tissue or EM grids (gift of Harry S. Vinters and Anne B. Johnson) used in previous publications [10] were examined in this study. Table 1 lists the six cases with an AD diagnosis ranging in age from 52 to 84 years as well as six non-AD control cases, aged 62–80 years. The non-AD control samples were of normal tissue obtained while performing surgery for encephalitis, hydrocephalus, or brain tumor. In addition to archived print images that were viewed using a JEOL 100CS electron microscope, a new set of digital EM images were captured on a Gatan US4000 4kx4k CCD camera for some of the cases. New sections were cut from the plastic embedded blocks. A large series of images was obtained from the parietal, prefrontal or frontal cortex and included images of neurons and glial cells with surrounding neuropil, and only the higher magnification images were used for this analysis where synaptic vesicles were clearly distinguishable.

**Table 1:** Cases used in the study

Case	Diagnosis	Age (yr)	Gender
AD-52	AD	52	F
AD-53	AD	53	M
AD-55	AD	55	M
AD-63	AD	63	M
AD-64	AD	64	M
AD-84	AD	84	F
C-62	Control	62	M
C-64	Control	64	M
C-69	Control	69	M
C-70	Control	70	n/a
C-74	Control	74	F
C-80	Control	80	M

### Image analysis

Image J software was used for quantification. For each image the scale was set according to the scale bar embedded in the image. Using classifications as previously described [8, 27] every presynaptic axonal bouton in the image was measured if it was membrane enclosed and contained a minimum of 10 synaptic vesicles. The area of each axonal bouton was measured. Then, using the counting tool, each vesicle with an identifiable membrane was counted. Each bouton was also classified as having only vesicles that were clear and round and of uniform size (35–40 nm), or as containing either enlarged vesicles (approximately 60 nm in diameter) and/or dense cored vesicles (often larger than 80 nm) [40]. When present, the mitochondria within each bouton were also analyzed and mitochondrial length, width, and area were measured. Each mitochondrion was classified as being either intact, where the membranes as well as the internal cristae structures were complete, or as being damaged, defined as having either the loss of outer membrane or loss of inner cristae structure. Each mitochondria data was kept associated with its specific presynaptic bouton so correlations between vesicle density and type and mitochondria parameters could be determined. The length of any post-synaptic density (PSD) associated with each bouton was also measured. Not all presynaptic axons were associated with a PSD and were classified as non-synaptic boutons [8]. For tripartite synaptic complexes with a presynaptic axon and a PSD, the dendritic spine was also similarly measured. The spines often contained mitochondria, spine apparatus, smooth ER, occasional microtubules, and no synaptic vesicles. The spine area and mitochondrial length, width, size, and type were recorded for each dendritic spine. The mitochondria within neurons were measured similarly. Table 2 lists the number of presynaptic axons, dendritic spines, neurons and mitochondria analyzed for this study.

**Table 2:** Number of synaptic compartments and mitochondria analyzed in this study

	AD cases	Control cases
# Presynaptic axons analyzed	480	442
# Presynaptic mitochondria analyzed	336	360
# Dendritic spines analyzed	213	162
# Spine mitochondria analyzed	42	35
# Neurons analyzed	16	15
# Neuronal soma mitochondria analyzed	333	439

A series of high magnification images of synapses with well-defined PSD regions and readily distinguishable vesicles docked at the active zone were selected to quantify the number of docked, recycling pool, and reserve pool vesicles. Representative synapses from the AD cases (n = 55) and the control cases (n = 31) were analyzed using a previously described method [61]. Briefly, for each synapse, starting at the active zone, compartments were delineated at every 80 nm interval outward, and the number of vesicles counted within each compartment. Docked vesicles, those in contact with the active zone, were counted separately. The recycling pool was considered to be those vesicles within 160 nm of the active zone. Vesicles outside the 160 nm compartment were classified as the reserve pool [61].

To evaluate the relative occurrence of the different sized vesicles in the AD and control groups, two representative boutons containing small round synaptic vesicles were randomly selected from each case and the diameter of all the vesicles measured within each bouton. The relative occurrence of each size group was determined for each case and the means for the AD and control groups determined.

### Statistical analysis

Statistical analysis was performed to compare either the means of each group (n = 6 per AD and control group) or between all structures in the AD and control groups combined. Student’s *t*-test, ANOVA, and regression analysis were adopted in differential testing and correlation analysis. We have made adjustments for multiple comparisons. Test statistics and significance levels were reported and the specific details were included in each figure legend. Graphpad Prism was used for preparing graphs and data analysis.

## Results

### Abnormal changes in the pre-synapse in the biopsied cortex from AD cases

**Fig. 1:**
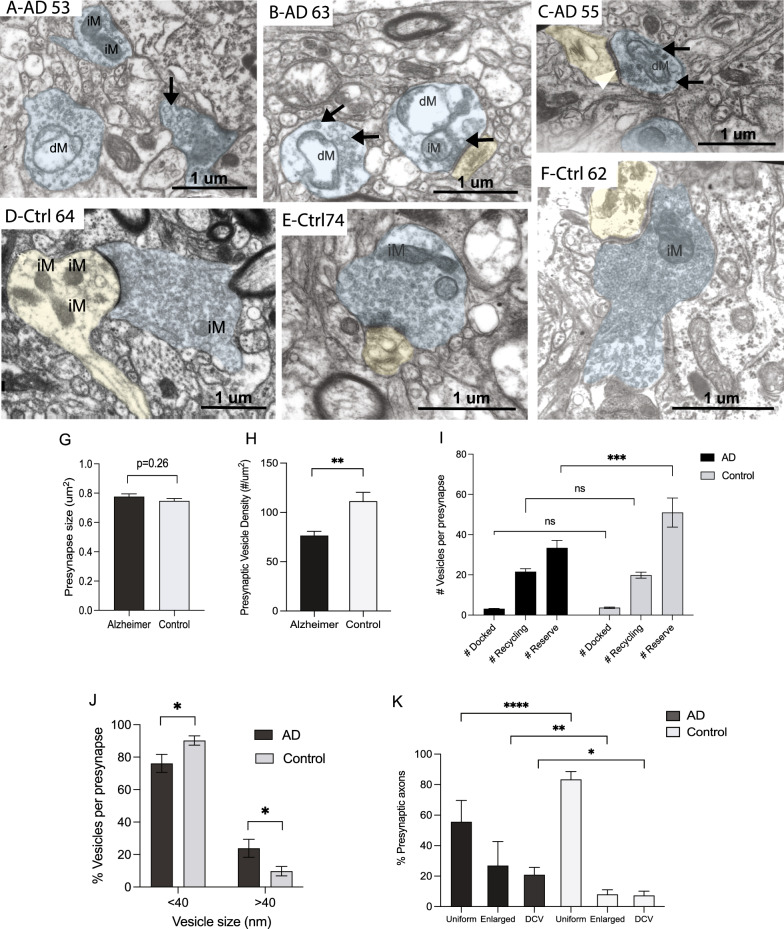
Representative EM images of presynaptic axon terminals from 3 different cases diagnosed with Alzheimer disease (**A**, **B**, **C**) and 3 different non-AD control cases (**D**, **E**, **F**). Presynaptic terminals are colored light blue and the dendritic spines are colored light yellow if present. Arrows mark enlarged or dense cored vesicles and white arrowhead marks a docked synaptic vesicle. While not every presynaptic axon terminal analyzed contained mitochondria, these images demonstrate both intact mitochondria (iM) and damaged mitochondria (dM). Case labels denote the age of the individuals and case details are listed in Table 1. No difference was noted in preysnapse size between the AD and control groups (G, *p* = 0.26). The AD cases have significantly fewer vesicles/µm^2^ axon area compared to non-AD cases (**H**, *p* < 0.01). There is no difference in the % of docked RRP vesicles between AD and control cases, however a greater % of vesicles are in the reserve pool in the control cases (**I**, *p* < 0.001). The AD presynapses have fewer vesicles with a diameter of 40 nm or less (**J**, *p* < 0.05) and have more vesicles larger than 40 nm (**J**, *p* < 0.05). The percent of presynaptic axonal boutons with either enlarged or dense cored vesicles is also higher in the AD cases compared to non-AD group (**K**, *p* < 0.0001). Data are Means ± S.E.M. of six AD and six control cases. Student t test (G, H, J) or one-way analysis of variance (**I**, **K**). *p < 0.05, **p < 0.01, ***p < 0.001, ****p < 0.0001

Electron micrographs of cortical tissues removed at biopsy from cases diagnosed with AD and age-matched non-AD control patients were analyzed for changes in synapses and synaptic mitochondria. In every case examined, synaptic complexes were clearly identifiable and mitochondria were found in subsets of presynaptic axonal boutons and postsynaptic dendritic spines (Fig. 1A–F). In the non-AD control samples, the majority of the presynaptic terminals contain clear round symmetrical synaptic vesicles (SVs) often filling up the entire axonal bouton (Fig. 1D–F, representative images from 3 cases). In AD cases, presynaptic axons with normal clear round SVs are also found, however, only a few presynaptic boutons were filled with clear, round uniform SVs, but rather many had large empty areas devoid of any structures (Fig. 1A-C, representative images from 3 cases). Also, within the presynaptic compartments, dense core vesicles and enlarged synaptic vesicles were more frequently seen in the AD cases (Fig. 1A–C, arrows), compared to the control samples.

A quantitative study of a total of 480 presynaptic boutons from six AD cases and 442 boutons from six age-matched control cases observed was completed. There was a slight increase in the mean size of presynaptic boutons in AD compared with non-AD controls, although it did not reach significance (*p* = 0.26, Fig. 1G). The mean SV density (number SVs per micron^2^ presynaptic area, less the area occupied by mitochondria when present) per case was determined and interestingly, the AD cases demonstrated a significantly lower SV density compared to the control cases (*p* < 0.01; Fig. 1H).

For each synapse, SVs can be classified into three pools, readily releasable pool (RRP, white arrowhead in Fig. 1B showing representative docked vesicle), recycling pool and reserve pool, according to their spatial distribution [61]. Images of tripartite synapses with apparent active zones from the AD cases (n = 55) and the control cases (n = 31), were further analyzed for vesicle distribution. There is no change of the RRP (number of docked vesicles per synapse) or the recycling pool between the AD and control synapses (Fig. 1I). However, the AD cases have a lower number of vesicles per synapse in the reserve pool (*p* < 0.001) compared to the control cases synapses (Fig. 1I).

Enlarged vesicles were more commonly seen in the AD images (Fig. 1A–C, arrows). To quantify potential changes in the size of SVs, two boutons were randomly selected from each case and the diameter of all the vesicles measured within each bouton. Normal synaptic vesicle size is reported to be 35–40 nm in diameter. The control case synapses had a higher relative percentage of smaller vesicles (40 nm or less, *p* < 0.05, Fig. 1J) and a lower percentage of the larger size vesicles compared to the AD group (greater than 40 nm, *p* < 0.05, Fig. 1J).

Each of the 480 AD presynaptic boutons and 442 control boutons were classified as having only clear uniformly sized vesicles or as having either enlarged vesicles or dense core vesicles and the % occurrence of each of these categories was determined for each case. The AD cases have significantly fewer presynaptic compartments with only clear round vesicles, about 56%, compared to 83% in the normal patient samples (*p* < 0.0001, Fig. 1K). Further, the AD cases had a higher percentage of boutons with enlarged vesicles, 27% vs 8% in the controls (*p* < 0.01) and a higher percentage of boutons with dense core vesicles at 21% vs 7% in controls (*p* = 0.05, Fig. 1K).

### Abnormal changes in the pre-synaptic mitochondria in the biopsied AD cortex

**Fig. 2:**
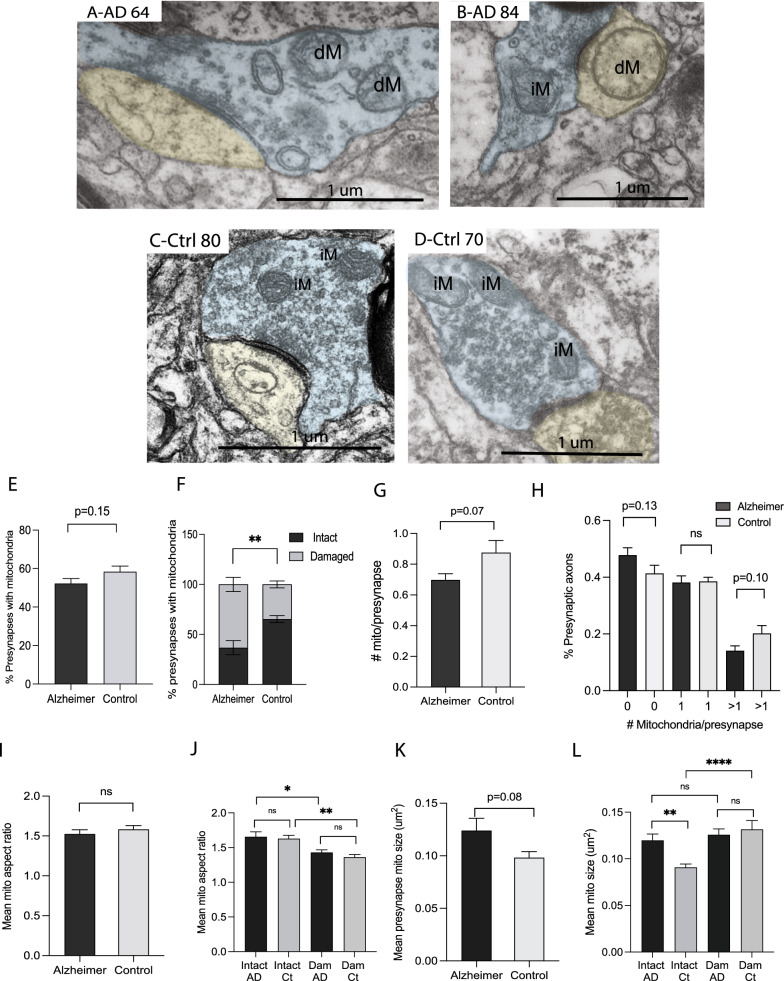
Representative EM images highlight damaged (dM) and intact mitochondria (iM) in AD (**A**, **B**) and non-AD axon terminals (**C**, **D**). Presynaptic terminals are colored light blue and the dendritic spines are colored light yellow if present. The control cases display a trend toward more presynapses with mitochondria (**E**, *p* = 0.15), however, the percentage of presynaptic axons with damaged mitochondria is significantly greater in the AD group (**F**, p < 0.01). The number of mitochondria per presynaptic axon is lower in AD cases (**G**, *p* = 0.07), and further analysis finds that the mean % of preysnapses lacking mitochondria trends higher in the AD group (*p* = 0.13), while the control cases have a higher % with more than one mitochondria (*p* = 0.10) (H). Mitochondria aspect ratio is not different between the AD and control cases (**I**, *p* > 0.05). Damaged mitochondria have lower aspect ratios than intact mito in both AD and non-AD groups (**J**, *p* < 0.05 AD, *p* < 0.01control group). Mitochondria size only trends higher in the AD cases (**K**, *p* = 0.08). Yet, intact mitochondria in AD presynaptic boutons are larger than those in non-AD cases (**L**, *p* < 0.01). While the mean size of AD mitochondria is unchanged between intact and damaged groups (**L**, *p* > 0.05), the damaged mitochondria in control cases are larger than their intact counterparts (**L**, *p* < 0.0001). Data are Means ± S.E.M. of six AD and six control cases. Student t test (**E**–**I**, **K**) or one-way analysis of variance (**J**, **L**). *p < 0.05, **p < 0.01, ****p < 0.0001

Mitochondria were found in around 59% of presynapses in control cases, but decreased to around 52% of presynapses in AD (Fig. 2E, *p* = 0.15). The mitochondria were classified as either intact mitochondria (iM) which exhibited a typical double-walled morphology with cristae spanning the mitochondria, or damaged mitochondria (dM) which appeared swollen or with damaged internal cristae structure or damaged membrane. Intact and damaged mitochondria are seen in the representative images of presynaptic axons from both AD cases (Fig. 2A, B) and control cases (Fig. 2C, D). In control cases, the majority of mitochondria found in the presynaptic axons are intact, while some damaged mitochondria, lacking either a clear outer membrane or inner membrane structure, were occasionally found. However, in AD cases, mitochondria often appeared round and swollen, and were classified as damaged with near total loss of cristae structure. Quantification revealed over 63% of the AD mitochondria present are damaged (average of cases ranged from 40 to 95%) compared to only 27% (average of cases ranged from 16 to 37%) in the non-AD group (*p* < 0.01; Fig. 2F).

All mitochondria were counted. The AD cases had fewer mitochondria per synapse, though this did not reach significance (*p* = 0.07, Fig. 2G). As a group, comparing the average occurrences per case, the AD cases had more axons lacking mitochondria, and fewer axons with more than one mitochondria than the controls, though this did not reach significance (*p* = 0.13 and *p* = 0.10, Fig. 2H).

Each mitochondrion was measured for length (long axis), width (short axis) and size (um^2^). From this data, the aspect ratio (long axis/short axis), a measure of mitochondrial roundness, was calculated. The presynaptic mitochondrial aspect ratio was slightly reduced in AD cases, although it did not reach significance (Fig. 2I), and the mean mitochondrial size was found to be larger in the AD group, though it did not reach significance (*p* = 0.08, Fig. 2K).

Morphological characteristics of the intact and damaged mitochondria were then further analyzed separately. Interestingly, damaged mitochondria have significantly decreased aspect ratios compared to intact mitochondria within both the AD (*p* < 0.05, Fig. 2J) and control groups (*p* < 0.01, Fig. 2J). However, no differences in aspect ratios are noted in the intact groups or damaged groups between the AD and control cases (Fig. 2J), suggesting the decreased aspect ratio in AD cases is largely due to increased percentage of damaged mitochondria in AD. We also compared the size of intact and damaged mitochondria separately and found that intact mitochondria in the AD cases are larger than intact presynaptic mitochondria in the control cases (*p* < 0.01; Fig. 2L). No significant difference was found in the size between intact and damaged mitochondria in the AD group. However, the non-AD control group have damaged mitochondria that are significantly larger than intact ones (*p* < 0.0001, Fig. 2L).

### Correlation between deficits in presynaptic mitochondria and pre-synapses in all cases

**Fig. 3:**
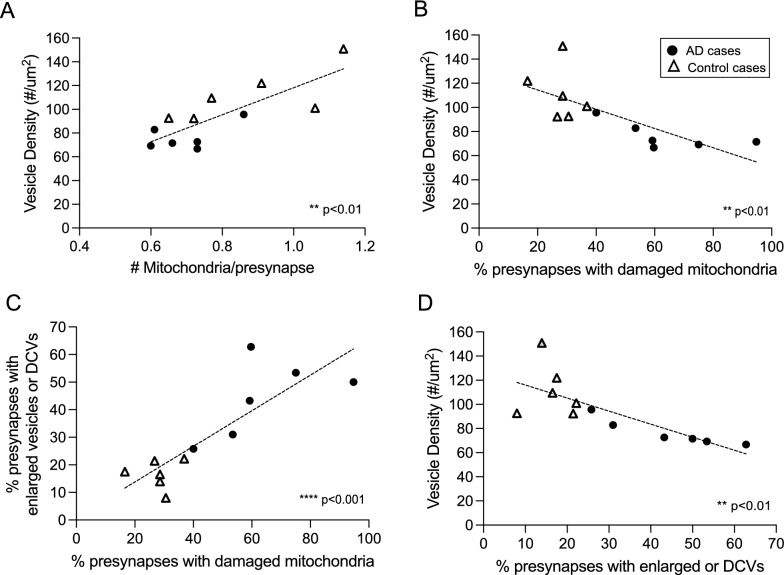
Correlation of mitochondrial changes with synaptic vesicle numbers and morphology in all cases. There is a strong correlation of the mean # of mitochondria per presynapse against mean vesicle density per case using regression analysis (**A**, *p* < 0.01). The prevalence of damaged mitochondria is negatively correlated with vesicle density (**B**, *p* < 0.01). The presence of enlarged vesicles and DCVs is correlated with damaged mitochondria (**C**, *p* < 0.001). A greater number of enlarged vesicles and DCVs is negatively correlated with vesicle density (**D**, *p* < 0.01). The AD cases (blue circles) often cluster as a group compared to the control cases (red circles). **p < 0.01, ***p < 0.001

Considering the importance of mitochondria for synaptic function, we performed correlation analysis between synaptic mitochondria and synaptic vesicles among all cases. First, synaptic vesicle density is positively correlated with the number of mitochondria in the presynaptic axons (Fig. 3A, **p < 0.01) but negatively correlated with the percentage of damaged mitochondria (Fig. 3B, **p < 0.01). In addition to the degree of mitochondria damage correlating with loss of synaptic vesicles, mitochondria damage is also correlated with increased occurrence of enlarged vesicles or dense cored vesicles in the presynaptic axons (Fig. 3C, ****p < 0.001). Finally, the numbers of presynapses with enlarged or dense core vesicles also strikingly correlates with reduced vesicle density (Fig. 3D, **p < 0.01). Interestingly, these correlations apply to both the normal cases (white triangles) and AD cases (dark circles), and the AD cases tend to group together, suggesting there may be critical thresholds rather than a continuous function for both mitochondria number and morphological changes as well as synaptic vesicle number and morphological changes that define AD progression.

### Abnormal changes in the post-synaptic dendrite mitochondria in the biopsied AD cortex

**Fig. 4:**
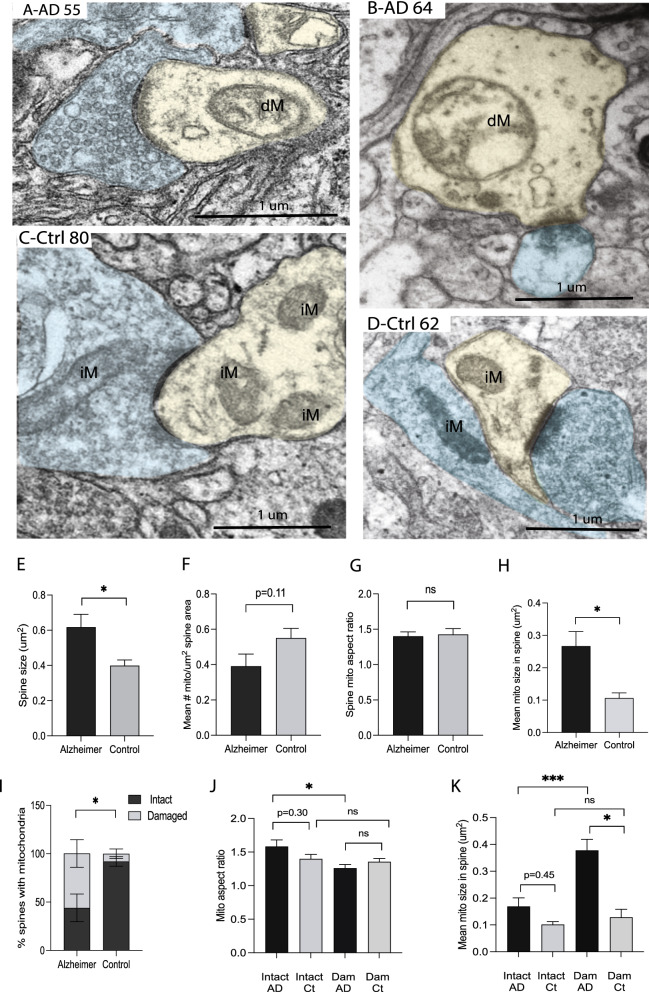
Representative EM images of dendritic spines containing mitochondria in AD cases (**A**, **B**) and control cases (**C**, **D**). Presynaptic terminals are colored light blue and the dendritic spines are colored light yellow. Damaged mitochondria are noted as (dM), intact mitochondria as (iM). The dendritic spine size is larger in the AD group (**E**, *p* < 0.05). The number of mitochondria in the dendritic spine is slightly decreased in the AD cases (*F*, *p* = 0.11), and there is no change in mean mitochondria aspect ratio between the AD and control cases (*G*, *p* > 0.05). However, mitochondria in post-synaptic dendrites find they are much larger in the AD cases (*H*, *p* < 0.05). The percentage of dendritic spines with damaged mitochondria is also higher in the AD cases compared to controls (*I*, *p* < 0.05). Comparison of the aspect ratio and size for the intact and damaged mitochondria separately, finds that the damaged mitochondria have lower aspect ratios than intact mitochondria in AD cases only (*J*, *p* < 0.05). In the AD cases, spine damaged mitochondria are larger than the intact mitochondria (*K*, *p* < 0.001), and are larger than the damaged mitochondria in control cases (*K*, *p* < 0.05). Data are Means ± S.E.M. of six AD and six control cases. Student t test (E-I) or one-way analysis of variance (**J**, **K**). *p < 0.05, ***p < 0.001

The same parameters were used to quantify the post-synaptic dendrites and the mitochondria contained within them. Only those dendrites that were both apposed to a presynaptic axonal bouton and contained a PSD were analyzed. No change in PSD length was noted between the AD and control cases in this dataset (data not shown). However, the size of spines was significantly increased in AD compared with non-AD controls (Fig. 4E, *p* < 0.05). Qualitatively, many of the dendritic spines in the AD cases had large areas devoid of any structures, and the mitochondria frequently appeared larger and rounder and had damaged internal cristae (Fig. 4A, B). In the control cases, the mitochondria were smaller with intact cristae (Fig. 4C, D). There was a trend towards reduced mean number of mitochondria per spine area (#/µm^2^) in the AD cases compared to that in the control cases, though this did not reach significance (*p* = 0.11, Fig. 4F). There was no difference in the aspect ratio of the spine mitochondria between the AD and control patient groups (Fig. 4G). Strikingly, however, the dendritic spine mitochondria in the AD cases are over 2.5 times larger than those in the controls (p < 0.05, Fig. 4H). Over 90% of dendritic mitochondria present in the post-synaptic dendrites in the non-AD control cases were classified as intact, with complete outer membrane and inner cristae structure. Compared to the less than 10% of the control case mitochondria that were damaged, in the AD cases over 60% of the mitochondria appear damaged in the dendritic spines (*p* < 0.05, Fig. 4I). Comparison of the aspect ratios separately for either the intact or the damaged mitochondria finds that damaged mitochondria have significantly decreased aspect ratios compared to intact mitochondria within the AD group (*p* < 0.05, Fig. 4J). No differences were seen comparing intact and damaged mitochondria aspect ratio in the control group which is likely due to the small number of damaged mitochondria found in controls cases (Fig. 4J). Within the AD cases, the mitochondria with a damaged morphology are significantly larger than those with intact morphology (*p* < 0.001, Fig. 4K). AD damaged mitochondria are larger than the control damaged mitochondria (*p* < 0.05, Fig. 4K).

### Abnormal changes in soma mitochondria in biopsied AD brain

**Fig. 5:**
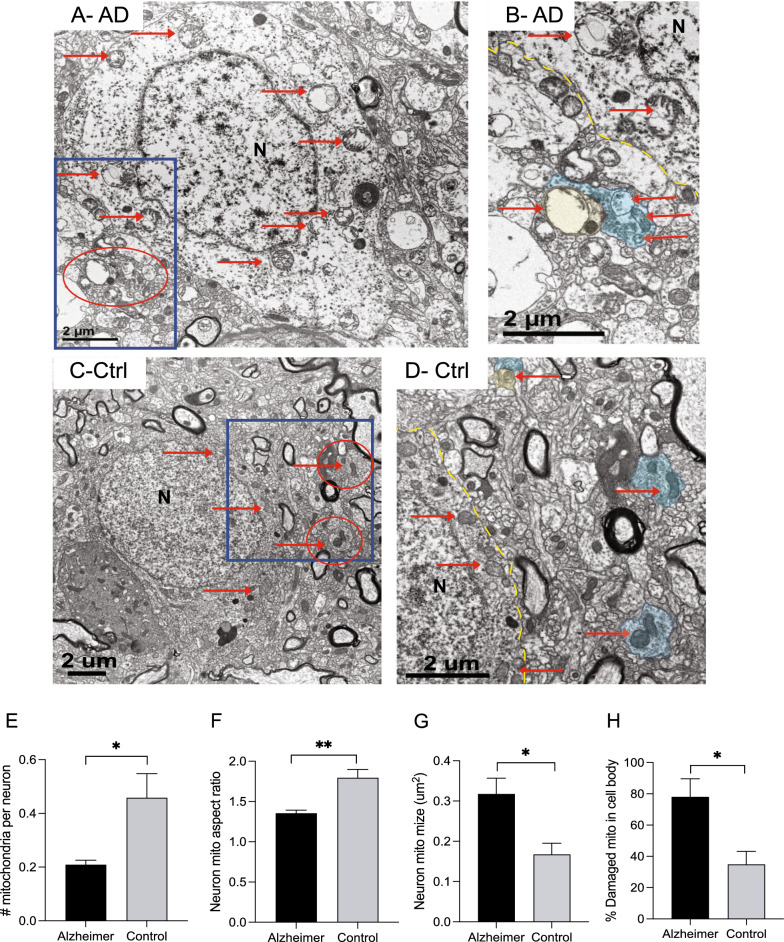
Representative EM images of neuronal cell bodies from an AD case (**A**) and control case (**C**). Enlarged images of the boxed regions show mitochondria in neurons and nearby synapses (**B**, **D**). Presynaptic terminals are colored light blue, the dendritic spines are colored light yellow, and some mitochondria are noted with red arrows. There are fewer mitochondria per neuronal area in the AD cases (**E**, *p* < 0.05). Morphological analysis finds that the mitochondria aspect ratio is lower in AD (**F**, *p* < 0.01) and the mean mitochondria is larger in AD (**G**, *p* < 0.05). On average, more mitochondria display a damaged morphology in the AD cases, compared to the control cases (**H**, *p* < 0.05). Data are Means ± S.E.M. of 16 AD neurons and 15 control neurons from six AD and six control cases. Student t test (**E**–**H**). *p < 0.05, **p < 0.01

For comparison, we also measured mitochondria in neuron cell bodies. A total of 16 neurons from the AD group and 15 neurons from the non-AD group were examined. Representative images of an AD case neuron (Fig. 5A, B is enlarged blue boxed area from A) and a control case neuron (Fig. 5C, D is enlarged blue boxed area from C) were shown. Enlarged regions show mitochondria in synapses and nearby neuronal soma, and the remarkable size differences in the mitochondria in the different compartments in cases of AD is apparent. The mean # of mitochondria/µm^2^ cytoplasmic area is 0.46 in control neurons compared to only 0.21 in the AD neurons (Fig. 5E, *p < 0.05). Mean mitochondria aspect ratio is significantly reduced in the AD cases compared to control neurons (Fig. 5F, *p* < 0.01). Mitochondria size is increased from 0.17 µm^2^ in the control neurons to 0.32 µm^2^ in the AD neurons (Fig. 5G, *p < 0.05). In addition, the vast majority of mitochondria in the neuron soma are damaged in the AD cases compared to the control cases (Fig. 5H, *p < 0.05).

Direct comparison of mitochondria in the three individual compartments (i.e., neuronal soma, presynaptic axonal bouton, and postsynaptic dendritic spine) finds that mitochondria number or coverage was decreased in all three compartments of AD brain, though it only reaches significance in the neuronal cell body (Additional file 1), suggesting impaired mitochondrial biogenesis may be involved [64]. Mitochondrial aspect ratio, as well, was significantly lower only in the AD neuronal cell bodies. However, the mean mitochondria size is significantly higher in AD soma and AD dendritic spines compared to controls. The mitochondria in the soma and dendrites are nearly identical in size, while the mitochondria in the presynaptic bouton are significantly smaller in all cases. Importantly, in all 3 compartments, the majority of AD mitochondria display a damaged morphology, suggesting potential mitophagy impairment [44].

## Discussion

A few studies looking at mitochondria characteristics in AD synapse to date have utilized autopsy tissue sample when postmortem changes can alter results of their qualitative mitochondrial and vesicle morphological changes [21, 49]. Moreover, it is known that analysis of end-stage autopsy samples showed more severe synapse loss than biopsy samples collected during disease course [19], therefore, it is critical to examine mitochondria abnormalities and synaptic vesicle changes in AD brain samples taken at biopsy to understand the role of mitochondria dysfunction in synaptic deficits early in the disease course and free of postmortem artifacts. In this study, we did a thorough examination of the appearance and characteristics of mitochondria in both the pre-synaptic and post-synaptic compartments in biopsied cortical tissues from AD patients and age-matched non-AD controls. The better preservation of cellular and organelle structures in biopsied tissues allow more detailed characterization of ultrastructural changes in synapse and mitochondria which confirmed findings from previous studies such as reduced presynaptic terminals with multiple mitochondria in AD on autopsied tissues [49] but further revealed some interesting new findings: (1) Synaptic vesicle density in presynapses was significantly decreased in AD which appeared largely due to significantly decreased reserve pool; (2) reduced mitochondria and significantly enlarged and increased damaged mitochondria were widely spread in both pre- and post-synapses in AD; (3) mitochondrial deficits correlated with reduced synaptic vesicles in AD pre-synapses.

A striking result documented in our study is the consistent decrease in synaptic vesicle density in the presynaptic axon terminals in AD cases. This is not due to a change in the axonal bouton itself since we found insignificant changes in bouton size, but rather it is the loss of the synaptic vesicles that discriminate the AD cases from the non-AD samples. While this was not quantified in prior EM studies on human autopsied brain tissues, it is consistent with prior studies in cell and animal models of AD: amyloid beta reduced synaptic vesicle stores in neuronal cultures [46] and hippocampal synaptic vesicle density decreased in the presynaptic terminals in APP/PS1, 5xFAD mice and 3xTg AD mice early during the course of disease [2, 11, 58], suggesting reduced synaptic vesicles is likely an early synaptic change in human AD. Interestingly, more detailed analysis of changes in different SV pools in our study revealed a significant reduction of reserve pool but insignificant changes in RRP and recycling pool, suggesting that the reduced SVs in AD is likely due to reduced reserve pool. Recent studies demonstrated that increased RRP mediated short-term memory in mossy fiber synapses and large reserve pool is needed to ensure fast and sustained reloading of the RRP [75]. Reduced reserve pool limited synaptic transmission during repetitive synaptic activity [50, 57] and an intact reserve pool is necessary for memory processing under challenging conditions [7]. Impaired learning and memory function was associated with reduced vesicle pool [48, 83]. Therefore, our finding demonstrated that reduced synaptic vesicles also contributed to synaptic deficits in AD, which provides strong support for the numerous biochemical studies that have reported loss of vesicle proteins in AD brain tissue [6, 15, 18, 30, 55, 70, 84]. It is of particular interest to note that, synapsin, a key protein involved in maintaining reserve pool of SVs, is among these reduced proteins [43, 54, 59]. Synapsin knockout caused age-dependent synaptic deficit and cognitive impairment in mice [14, 53], suggesting that reduced synapsin expression could contribute to the synaptic deficits we observed in AD although its specific role in AD needs to be further elucidated.

In contrast to reduced SV proteins in AD brain, it has been known for a while that the dense core vesicle specific protein chromogranin A is increased in AD brain [37, 38]. In fact, the significant increase of the ratios of chromogranin A to SV markers such as synaptophysin, p65 or SV2 in AD were significantly correlated to clinical severity of dementia [38] which prompted a speculation that distinct changes occur for SV and DCVs in AD with the caveat that DCV proteins are also present in the soma and their accumulation in AD may not necessarily reflect changes of DCVs in presynapse. Interestingly, while less than 5% of the presynaptic axons from non-AD cases contain dense core vesicles, there was significantly increased occurrence of DCV vesicles in over 20% of the presynaptic axons in AD cases. Our finding of increased DCVs in contrast to reduced SVs in presynapses in AD thus provided direct evidence supporting the notion of distinct changes between SVs and DCVs in AD presynapses. DCV accumulation in AD presynapse is likely due to defects in DCV release because neuronal secretion of DCV proteins is impaired by Aβ in vitro and in situ [51]. Chronic neuronal inactivity also blocked DCV release and resulted in DCVs accumulation in the pre-synapse [73]. The failure in DCV release in AD is consistent with previous observation of aberrant accumulation of DCV proteins in dystrophic neurites, degenerative structures originated from axons [35, 52, 82] and decreased DCV proteins in the CSF as a potential biomarker for AD [5]. Because proper DCV release is important for peptidergic transmission which controls circuitry function and homeostasis [24], our finding suggests that alterations in peptidergic transmission is likely involved in AD.

Another major finding of the current study is the reduced total number of mitochondria along with significantly increased number of damaged mitochondria in the presynapses in AD biopsy samples. The percentage of presynapses with mitochondria decreased from 59 to 52% and the average number of mitochondria per presynapse also decreased in AD. Our study confirmed a trend towards reduction in the percentage of presynaptic terminals with multiple mitochondria in AD brain as previously reported in the autopsied brain [49], but also identified a trend towards increase in the percentage of presynaptic terminals without any mitochondria in AD. In control presynapses, the majority of mitochondria appear normal with intact cristae and membranes and were often shorter and thinner than those in the soma. Enlarged mitochondria were commonly seen in AD with significant number of mitochondria displaying broken cristae or membranes, demonstrating significant mitochondrial damage in AD synapses which likely indicated mitochondrial dysfunction. Since both intact and damaged mitochondria can often be seen in the same region, mitochondrial damage is unlikely an artifact of tissue preparation. Decreased aspect ratio of damaged mitochondria in both AD and control pre-synapse comparing to the intact mitochondria in control presynapses indicated abnormal mitochondria dynamics towards unbalanced fission and their increased size was consistent with a swollen appearance. It is worth noting that the protein expression of mitochondrial fission and fusion proteins were altered in AD which likely caused mitochondrial fragmentation in AD [80], resulting in increased oxidative stress and reduced calcium buffering capacity. In fact, unbalanced mitochondrial fission caused mitochondrial damage and swelling that eventually led to neurodegeneration in the cortex and hippocampus in mice [26, 31]. On the other hand, intact mitochondria in AD pre-synapse also demonstrated significantly increased size compared to that of control pre-synapse. This may be a compensatory response to reduced mitochondrial function in AD pre-synapse since these mitochondria still maintained their fission/fusion balance (i.e., aspect ratio), however, the possibility that these intact mitochondria in AD pre-synapse may be at initial stage of mitochondrial damage could not be ruled out. Our study thus provided strong support of increased presynaptic mitochondrial damage in AD which is consistent with well documented synaptic mitochondrial dysfunction in the brain of AD patients [23, 28]. Furthermore, similar synaptic mitochondrial deficits were reported in cell and animal models of AD [32]: Aβ treatment of synaptosome caused mitochondrial dysfunction [36] as well as swollen synaptic mitochondria along with significantly reduced number of SVs [45]; Significantly reduced presynaptic mitochondria [63] and increase in presynaptic mitochondria lacking cristae and bioenergetics deficits along with reduced SVs were found in 5xFAD mice [2]. Mitochondria are strategically localized to presynaptic sites to meet the local needs and the proximity of mitochondria influences synaptic vesicle number [67]. Reduced presynaptic mitochondria or mitochondrial inhibition led to reduced SV pool [66, 85], either through reduced ATP provision or interrupted calcium buffering. Indeed, there was a significant positive correlation between synaptic vesicle density and the number of mitochondria in the presynaptic axons and a negative correlation between synaptic vesicle density and the percentage of damaged mitochondria in the presynapse of AD and control, which suggest that both reduced mitochondria and increased mitochondrial damage contribute to synaptic deficits in AD.

Mitochondria were much less abundant in postsynaptic spines than in presynapses [49]. Similar mitochondrial deficits including reduced mitochondria density, reduced number of spines containing mitochondria and significantly enlarged and increased damaged mitochondria were also found in AD spines in our study. Prior studies demonstrated alterations of spine morphology in AD brain [3] but we only found significantly increased spine size in AD which could be account for decreased mitochondrial density in AD spines containing mitochondria. Nevertheless, reduced number of spines containing mitochondria and increased damaged mitochondria in the spine could still contribute to synaptic dysfunction such as impaired LTP induction and memory formation which requires functional mitochondria capable of fission and calcium handling [20].

Collectively, these data demonstrated reduced mitochondria along with widespread mitochondrial damage in both presynaptic axon terminals and postsynaptic spines in cortical tissues in AD, suggesting that mitochondrial dysfunction in both pre- and postsynaptic compartments contributes to synaptic deficits in AD.

## Supplementary Information

**Additional file 1**: Comparison of mitochondria in pre-synapse, post-synapse and neuron compartments.

## Data Availability

All data are available upon reasonable request to the corresponding authors.
