# Supplementary material for: Damaged mitochondria coincide with presynaptic vesicle loss and abnormalities in alzheimer’s disease brain
Source: Acta Neuropathol Commun. 2023 Mar 31;11:54. doi: 10.1186/s40478-023-01552-7 (PMC10067183; doi:10.1186/s40478-023-01552-7)

Electronic Supplementary Material

Acta Neuropathologica

Damaged Mitochondria Coincide with Presynaptic Vesicle Loss and Abnormalities in Alzheimer's Disease Brain

Wenzhang Wang[1*], Fanpeng Zhao[1*], Yubing Lu[1], Sandra L. Siedlak[1], Hisashi Fujioka[2], Hao Feng[3], George Perry[4], Xiongwei Zhu[1]

[1]Department of Pathology, Case Western Reserve University, Cleveland, OH, USA
[2]Cryo-EM Core Facility, Case Western Reserve University, Cleveland, OH, USA
[3]Department of Population and Quantitative Health Sciences, Case Western Reserve University, Cleveland, OH, USA
[4]Department of Neuroscience, Developmental and Regenerative Biology, University of Texas, San Antonio, San Antonio, TX, USA
*equal contribution

Correspondence to:
Xiongwei Zhu, PhD, Department of Pathology, Case Western Reserve University, 2103 Cornell Road, Cleveland, OH 44106, USA.
Tel.: 216-368-5903; E-mail: xiongwei.zhu@case.edu

Online Resource 1. Direct comparison of the mitochondria changes in the presynapse, post synaptic dendritic spines, and neuronal cell bodies. In AD, all 3 compartments have fewer mitochondria than the control cases, but only in neurons is this significant (A). Also, only in neurons, is the aspect ratio significantly decreased (B). However, the mean mitochondrial size is 2-3 fold higher in both the neurons and dendritic spines in the AD cases (C). The numbers of damaged mitochondria is a feature of all three compartments (D).

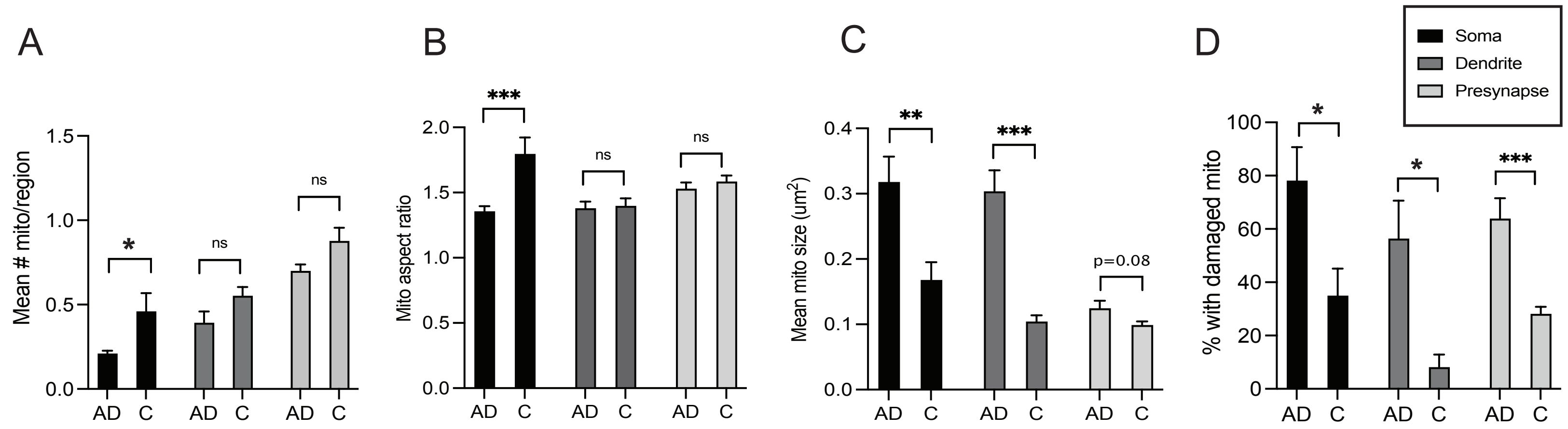

Supplement: Supplementary file 1 — Additional file 1: Comparison of mitochondria in pre-synapse, post-synapse and neuron compartments. [file 40478_2023_1552_MOESM1_ESM.pdf]
